# 5-(3-Methoxy­pheneth­yl)-4-(2-methoxy­phen­yl)-4*H*-1,2,4-triazol-3-ol

**DOI:** 10.1107/S1600536808033990

**Published:** 2008-10-25

**Authors:** Muhammad Hanif, Ghulam Qadeer, Nasim Hasan Rama, Wai-Yeung Wong

**Affiliations:** a Department of Chemistry, Quaid-I-Azam University, Islamabad 45320, Pakistan; b Department of Chemistry, Hong Kong Baptist University, Waterloo Road, Kowloon Tong, Hong Kong

## Abstract

In the mol­ecule of the title compound, C_18_H_19_N_3_O_3_, the triazole ring is oriented with respect to the 3-methoxy­phenyl and 2-methoxy­phenyl rings at dihedral angles of 11.79 (3) and 89.22 (3)°, respectively. The dihedral angle between the two benzene rings is 85.95 (3)°. In the crystal structure, inter­molecular O—H⋯N and C—H⋯O hydrogen bonds link the mol­ecules. There is a π–π contact between the triazole and 3-methoxy­phenyl rings [centroid–centroid distance = 3.916 (3) Å]. There is a π–π contact between the triazole and one of the 3-methoxy­phenyl rings [centroid–centroid distance = 3.916 (3) Å ]. C—H⋯π contacts are also found between the benzene ring and the methyl groups of their 3-methoxy-substituents.

## Related literature

For general background, see: Demirbas *et al.* (2002 ▶); Holla *et al.* (1998 ▶); Kritsanida *et al.* (2002 ▶); Omar *et al.* (1986 ▶); Paulvannan *et al.* (2000 ▶); Turan-Zitouni *et al.* (1999 ▶). For related structures, see: Öztürk *et al.* (2004*a* ▶,*b* ▶). For bond-length data, see: Allen *et al.* (1987 ▶).
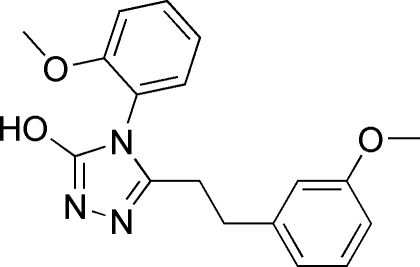


## Experimental

### 

#### Crystal data

C_18_H_19_N_3_O_3_
*M* *_r_* = 325.36
Monoclinic, 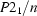

*a* = 10.5030 (11) Å
*b* = 14.1172 (14) Å
*c* = 11.3226 (11) Å
β = 98.192 (2)°
*V* = 1661.7 (3) Å^3^
*Z* = 4
Mo *K*α radiation
μ = 0.09 mm^−1^
*T* = 294 (2) K
0.32 × 0.24 × 0.22 mm

#### Data collection

Bruker SMART CCD diffractometer
Absorption correction: multi-scan (*SADABS*; Sheldrick, 1996 ▶) *T*_min_ = 0.902, *T*_max_ = 1.000 (expected range = 0.884–0.980)
9949 measured reflections
4026 independent reflections
3212 reflections with *I* > 2σ(*I*)
*R*_int_ = 0.018

#### Refinement

*R*[*F* ^2^ > 2σ(*F* ^2^)] = 0.048
*wR*(*F* ^2^) = 0.146
*S* = 1.02
4026 reflections
218 parameters
H-atom parameters constrained
Δρ_max_ = 0.56 e Å^−3^
Δρ_min_ = −0.40 e Å^−3^

### 

Data collection: *SMART* (Bruker, 1998 ▶); cell refinement: *SAINT* (Bruker, 1999 ▶); data reduction: *SAINT*; program(s) used to solve structure: *SHELXS97* (Sheldrick, 2008 ▶); program(s) used to refine structure: *SHELXL97* (Sheldrick, 2008 ▶); molecular graphics: *ORTEP-3 for Windows* (Farrugia, 1997 ▶) and *PLATON* (Spek, 2003 ▶); software used to prepare material for publication: *SHELXTL* (Sheldrick, 2008 ▶) and *PLATON*.

## Supplementary Material

Crystal structure: contains datablocks I, global. DOI: 10.1107/S1600536808033990/hk2549sup1.cif

Structure factors: contains datablocks I. DOI: 10.1107/S1600536808033990/hk2549Isup2.hkl

Additional supplementary materials:  crystallographic information; 3D view; checkCIF report

## Figures and Tables

**Table 1:** Hydrogen-bond geometry (Å, °)

*D*—H⋯*A*	*D*—H	H⋯*A*	*D*⋯*A*	*D*—H⋯*A*
O3—H3⋯N3^i^	0.82	1.94	2.7569 (15)	173
C5—H5*A*⋯O1^ii^	0.93	2.59	3.406 (2)	147
C8—H8*A*⋯O2	0.97	2.57	3.485 (2)	157
C4—H4*A*⋯*Cg*3^iii^	0.93	3.25	4.004 (3)	140
C7—H7*A*⋯*Cg*3	0.93	3.16	4.067 (3)	165
C18—H18*A*⋯*Cg*2^iv^	0.96	3.03	3.400 (3)	105
C18—H18*B*⋯*Cg*2^iv^	0.96	3.08	3.400 (3)	101

Symmetry codes: (i) 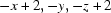; (ii) 
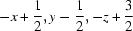
; (iii) 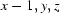; (iv) 
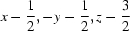
. *Cg*2 and *Cg*3 are the centroids of the C2–C7 and C C12–C17 rings, respectively.

**Fig. 1.**
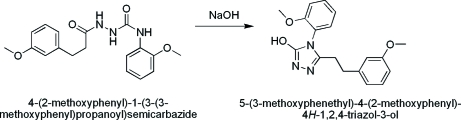
The formation of the title compound.

**Fig. 2.**
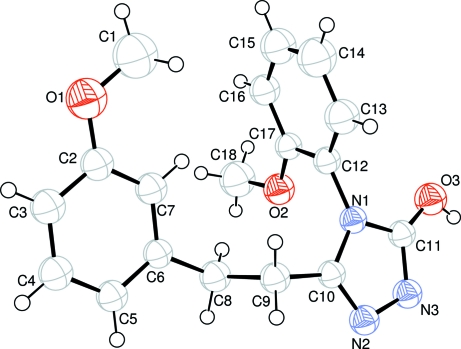
The molecular structure of the title molecule, with the atom-numbering scheme.

**Fig. 3.**
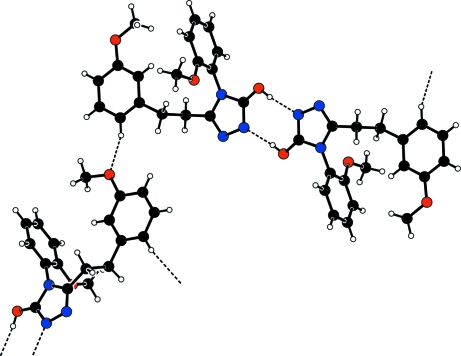
A partial packing diagram. Hydrogen bonds are shown as dashed lines.

## supplementary crystallographic information

### Comment

Substituted triazole derivatives display significant biological activities including antimicrobial (Holla *et al.*,  1998), analgesic (Turan-Zitouni *et al.*, 1999), antitumor (Demirbas *et al.*,  2002), antihypertensive (Paulvannan *et al.*,  2000) and antiviral (Kritsanida *et al.*,  2002) activities. The biological activity is closely related to the structure, possibly being due to the presence of the —N—C—S unit (Omar *et al.*,  1986). We are interested in the syntheses and biological activities of the aryloxyacetyl hydrazide derivatives and report herein the synthesis (Fig. 1) and crystal structure of the title compound.

In the molecule of the title compound (Fig. 2), the bond lengths (Allen *et al.*,  1987) and angles are within normal ranges, and they are comparable with those observed in related structures (Öztürk *et al.*, 2004*a*, 2004*b*). In the triazole ring, the N3=C11 [1.3459 (17) Å] bond has double bond character. Rings A (C2-C7), B (N1/N2/N3/C10/C11) and C (C12-C17) are, of course, planar and the dihedral angles between them are A/B = 11.79 (3)°, A/C = 89.22 (3)° and B/C = 85.95 (3)°.

In the crystal structure, intramolecular C-H···O and intermolecular O-H···N and C-H···O hydrogen bonds (Table 1) link the molecules (Fig. 3), in which they may be effective in the stabilization of the structure. The π—π contact between the triazole and 3-methoxyphenyl rings, Cg1···Cg2^i^ [symmetry code: (i) 1/2 + x, 1/2 - y, 1/2 + z, where Cg1 and Cg2 are the centroids of the rings B (N1/N2/N3/C10/C11) and A (C2-C7), respectively] may further stabilize the structure, with centroid-centroid distance of 3.916 (3) Å. There also exist C—H···π contacts (Table 1) between the phenyl rings and the methyl group and the 3-methoxyphenyl ring.

### Experimental

The synthesis of the title compound (Fig. 1) was carried out by refluxing a solution of 4-(2-methoxyphenyl)-1-(3-(3-methoxyphenyl)propanoyl)semicarbazide (3.43 g, 10 mmol) in NaOH (2M) for 5 h. Single crystals suitable for X-ray analysis were obtained by recrystallization from an aqeous ethanol solution at room temperature (yield; 71%, m.p. 454-455 K).

### Refinement

H atoms were positioned geometrically, with O-H = 0.82 Å (for OH) and C-H = 0.93, 0.97 and 0.96 Å for aromatic, methylene and methyl H, respectively, and constrained to ride on their parent atoms with U_iso_(H) = xU_eq_(C,O), where x = 1.2 for aromatic and methylene H and x = 1.5 for all other H atoms.

### Crystal data

**Table:**

C_18_H_19_N_3_O_3_	*F*(000) = 688
*M**_r_* = 325.36	*D*_x_ = 1.301 Mg m^−^^3^
Monoclinic, *P*2_1_/*n*	Melting point: 454(1) K
Hall symbol: -P 2yn	Mo *K*α radiation, λ = 0.71073 Å
*a* = 10.5030 (11) Å	Cell parameters from 9949 reflections
*b* = 14.1172 (14) Å	θ = 2.4–28.3°
*c* = 11.3226 (11) Å	µ = 0.09 mm^−^^1^
β = 98.192 (2)°	*T* = 294 K
*V* = 1661.7 (3) Å^3^	Block, yellow
*Z* = 4	0.32 × 0.24 × 0.22 mm

### Data collection

**Table:**

Bruker SMART CCD diffractometer	4026 independent reflections
Radiation source: fine-focus sealed tube	3212 reflections with *I* > 2σ(*I*)
graphite	*R*_int_ = 0.018
φ and ω scans	θ_max_ = 28.3°, θ_min_ = 2.4°
Absorption correction: multi-scan (SADABS; Sheldrick, 1996)	*h* = −8→14
*T*_min_ = 0.902, *T*_max_ = 1.000	*k* = −18→18
9949 measured reflections	*l* = −14→14

### Refinement

**Table:**

Refinement on *F*^2^	Primary atom site location: structure-invariant direct methods
Least-squares matrix: full	Secondary atom site location: difference Fourier map
*R*[*F*^2^ > 2σ(*F*^2^)] = 0.048	Hydrogen site location: inferred from neighbouring sites
*wR*(*F*^2^) = 0.146	H-atom parameters constrained
*S* = 1.02	*w* = 1/[σ^2^(*F*_o_^2^) + (0.0854*P*)^2^ + 0.2782*P*] where *P* = (*F*_o_^2^ + 2*F*_c_^2^)/3
4026 reflections	(Δ/σ)_max_ < 0.001
218 parameters	Δρ_max_ = 0.56 e Å^−^^3^
0 restraints	Δρ_min_ = −0.40 e Å^−^^3^

### Special details

**Table:**

Geometry. All e.s.d.'s (except the e.s.d. in the dihedral angle between two l.s. planes) are estimated using the full covariance matrix. The cell e.s.d.'s are taken into account individually in the estimation of e.s.d.'s in distances, angles and torsion angles; correlations between e.s.d.'s in cell parameters are only used when they are defined by crystal symmetry. An approximate (isotropic) treatment of cell e.s.d.'s is used for estimating e.s.d.'s involving l.s. planes.
Refinement. Refinement of *F*^2^ against ALL reflections. The weighted *R*-factor *wR* and goodness of fit *S* are based on *F*^2^, conventional *R*-factors *R* are based on *F*, with *F* set to zero for negative *F*^2^. The threshold expression of *F*^2^ > σ(*F*^2^) is used only for calculating *R*-factors(gt) *etc*. and is not relevant to the choice of reflections for refinement. *R*-factors based on *F*^2^ are statistically about twice as large as those based on *F*, and *R*- factors based on ALL data will be even larger.

### Fractional atomic coordinates and isotropic or equivalent isotropic displacement parameters (Å^2^)

**Table:**

	*x*	*y*	*z*	*U*_iso_*/*U*_eq_	
O1	0.34683 (13)	0.50242 (9)	0.80167 (14)	0.0726 (4)	
O2	0.76819 (12)	0.19899 (8)	0.72362 (9)	0.0538 (3)	
O3	0.99935 (9)	0.12807 (7)	0.96442 (12)	0.0554 (3)	
H3	1.0436	0.0800	0.9720	0.083*	
N1	0.78261 (10)	0.16861 (7)	0.95758 (10)	0.0367 (2)	
N2	0.70357 (11)	0.02988 (8)	1.00087 (11)	0.0439 (3)	
N3	0.83555 (11)	0.02434 (8)	0.99977 (11)	0.0439 (3)	
C1	0.4774 (2)	0.53003 (16)	0.8160 (3)	0.0908 (8)	
H1A	0.4829	0.5979	0.8133	0.136*	
H1B	0.5180	0.5033	0.7529	0.136*	
H1C	0.5200	0.5077	0.8915	0.136*	
C2	0.31961 (15)	0.40769 (11)	0.80274 (13)	0.0485 (3)	
C3	0.18992 (15)	0.38405 (12)	0.79300 (14)	0.0525 (4)	
H3A	0.1277	0.4313	0.7837	0.063*	
C4	0.15417 (14)	0.29079 (12)	0.79721 (14)	0.0512 (4)	
H4A	0.0675	0.2751	0.7909	0.061*	
C5	0.24656 (13)	0.21925 (11)	0.81085 (13)	0.0447 (3)	
H5A	0.2216	0.1563	0.8151	0.054*	
C6	0.37517 (13)	0.24212 (10)	0.81805 (12)	0.0401 (3)	
C7	0.41200 (14)	0.33670 (11)	0.81434 (13)	0.0463 (3)	
H7A	0.4986	0.3523	0.8196	0.056*	
C8	0.47827 (14)	0.16657 (11)	0.83435 (14)	0.0480 (3)	
H8A	0.5424	0.1807	0.7831	0.058*	
H8B	0.4400	0.1059	0.8100	0.058*	
C9	0.54420 (13)	0.15956 (10)	0.96390 (13)	0.0440 (3)	
H9A	0.5505	0.2225	0.9987	0.053*	
H9B	0.4913	0.1214	1.0090	0.053*	
C10	0.67491 (12)	0.11732 (9)	0.97478 (11)	0.0378 (3)	
C11	0.88697 (13)	0.10760 (9)	0.97304 (12)	0.0390 (3)	
C12	0.79093 (12)	0.26338 (9)	0.91477 (12)	0.0374 (3)	
C13	0.80818 (19)	0.33857 (12)	0.99299 (15)	0.0583 (4)	
H13A	0.8117	0.3284	1.0746	0.070*	
C14	0.8202 (2)	0.42943 (12)	0.94939 (19)	0.0751 (6)	
H14A	0.8323	0.4805	1.0017	0.090*	
C15	0.8143 (2)	0.44367 (11)	0.82921 (18)	0.0652 (5)	
H15A	0.8226	0.5048	0.8006	0.078*	
C16	0.79629 (15)	0.36961 (11)	0.74987 (14)	0.0504 (4)	
H16A	0.7918	0.3806	0.6684	0.061*	
C17	0.78478 (12)	0.27786 (9)	0.79249 (12)	0.0386 (3)	
C18	0.7433 (3)	0.21156 (16)	0.59719 (16)	0.0767 (6)	
H18A	0.7335	0.1508	0.5590	0.115*	
H18B	0.8140	0.2447	0.5708	0.115*	
H18C	0.6659	0.2477	0.5769	0.115*	

### Atomic displacement parameters (Å^2^)

**Table:**

	*U*^11^	*U*^22^	*U*^33^	*U*^12^	*U*^13^	*U*^23^
O1	0.0682 (8)	0.0439 (6)	0.1023 (10)	0.0042 (5)	0.0004 (7)	0.0152 (6)
O2	0.0795 (8)	0.0433 (5)	0.0401 (5)	0.0046 (5)	0.0140 (5)	0.0015 (4)
O3	0.0367 (5)	0.0395 (5)	0.0911 (8)	0.0079 (4)	0.0121 (5)	0.0164 (5)
N1	0.0359 (5)	0.0333 (5)	0.0410 (5)	0.0062 (4)	0.0063 (4)	0.0081 (4)
N2	0.0369 (6)	0.0378 (6)	0.0574 (7)	0.0038 (5)	0.0083 (5)	0.0089 (5)
N3	0.0366 (6)	0.0354 (6)	0.0596 (7)	0.0049 (4)	0.0067 (5)	0.0105 (5)
C1	0.0816 (15)	0.0595 (11)	0.1215 (19)	−0.0176 (10)	−0.0199 (13)	0.0270 (12)
C2	0.0502 (8)	0.0443 (7)	0.0498 (8)	0.0048 (6)	0.0030 (6)	0.0096 (6)
C3	0.0448 (8)	0.0574 (9)	0.0541 (8)	0.0171 (7)	0.0023 (6)	0.0060 (7)
C4	0.0336 (7)	0.0659 (10)	0.0532 (8)	0.0044 (6)	0.0030 (6)	0.0014 (7)
C5	0.0388 (7)	0.0483 (7)	0.0459 (7)	−0.0014 (6)	0.0020 (5)	−0.0004 (6)
C6	0.0360 (6)	0.0455 (7)	0.0381 (6)	0.0050 (5)	0.0025 (5)	−0.0013 (5)
C7	0.0360 (7)	0.0498 (8)	0.0527 (8)	0.0015 (6)	0.0055 (6)	0.0075 (6)
C8	0.0408 (7)	0.0482 (8)	0.0536 (8)	0.0086 (6)	0.0022 (6)	−0.0071 (6)
C9	0.0372 (7)	0.0454 (7)	0.0508 (7)	0.0086 (5)	0.0106 (6)	0.0068 (6)
C10	0.0355 (6)	0.0390 (6)	0.0392 (6)	0.0041 (5)	0.0066 (5)	0.0066 (5)
C11	0.0361 (6)	0.0347 (6)	0.0461 (7)	0.0064 (5)	0.0053 (5)	0.0075 (5)
C12	0.0374 (6)	0.0317 (6)	0.0435 (7)	0.0055 (5)	0.0070 (5)	0.0069 (5)
C13	0.0829 (12)	0.0440 (8)	0.0467 (8)	0.0023 (8)	0.0046 (8)	−0.0013 (6)
C14	0.1112 (16)	0.0392 (8)	0.0729 (12)	−0.0050 (9)	0.0063 (11)	−0.0075 (8)
C15	0.0784 (12)	0.0353 (7)	0.0836 (12)	−0.0019 (7)	0.0172 (9)	0.0135 (8)
C16	0.0552 (8)	0.0438 (7)	0.0547 (8)	0.0058 (6)	0.0159 (7)	0.0167 (6)
C17	0.0380 (6)	0.0356 (6)	0.0435 (7)	0.0057 (5)	0.0102 (5)	0.0057 (5)
C18	0.1174 (18)	0.0713 (12)	0.0423 (9)	0.0071 (12)	0.0147 (10)	0.0014 (8)

### Geometric parameters (Å, °)

**Table:**

O3—H3	0.8200	C9—H9B	0.9700
N2—N3	1.3902 (16)	C10—N2	1.2946 (17)
C1—O1	1.413 (3)	C10—N1	1.3801 (17)
C1—H1A	0.9600	C11—O3	1.2325 (16)
C1—H1B	0.9600	C11—N3	1.3459 (17)
C1—H1C	0.9600	C11—N1	1.3854 (16)
C2—O1	1.3680 (19)	C12—C13	1.378 (2)
C2—C7	1.388 (2)	C12—C17	1.3919 (18)
C2—C3	1.391 (2)	C12—N1	1.4299 (15)
C3—C4	1.372 (2)	C13—C14	1.387 (2)
C3—H3A	0.9300	C13—H13A	0.9300
C4—C5	1.394 (2)	C14—C15	1.368 (3)
C4—H4A	0.9300	C14—H14A	0.9300
C5—C6	1.3798 (19)	C15—C16	1.374 (3)
C5—H5A	0.9300	C15—H15A	0.9300
C6—C7	1.393 (2)	C16—C17	1.3934 (19)
C6—C8	1.5123 (19)	C16—H16A	0.9300
C7—H7A	0.9300	C17—O2	1.3563 (17)
C8—C9	1.533 (2)	C18—O2	1.429 (2)
C8—H8A	0.9700	C18—H18A	0.9600
C8—H8B	0.9700	C18—H18B	0.9600
C9—C10	1.4857 (18)	C18—H18C	0.9600
C9—H9A	0.9700		
			
C2—O1—C1	118.00 (14)	H8A—C8—H8B	107.9
C17—O2—C18	117.66 (13)	C10—C9—C8	113.03 (11)
C11—O3—H3	109.5	C10—C9—H9A	109.0
C10—N1—C11	107.79 (10)	C8—C9—H9A	109.0
C10—N1—C12	129.03 (10)	C10—C9—H9B	109.0
C11—N1—C12	122.62 (11)	C8—C9—H9B	109.0
C10—N2—N3	104.56 (11)	H9A—C9—H9B	107.8
C11—N3—N2	112.67 (11)	N2—C10—N1	111.34 (11)
O1—C1—H1A	109.5	N2—C10—C9	125.71 (12)
O1—C1—H1B	109.5	N1—C10—C9	122.95 (11)
H1A—C1—H1B	109.5	O3—C11—N3	129.95 (12)
O1—C1—H1C	109.5	O3—C11—N1	126.41 (12)
H1A—C1—H1C	109.5	N3—C11—N1	103.64 (11)
H1B—C1—H1C	109.5	C13—C12—C17	120.63 (12)
O1—C2—C7	124.22 (15)	C13—C12—N1	120.78 (13)
O1—C2—C3	115.93 (14)	C17—C12—N1	118.57 (12)
C7—C2—C3	119.85 (14)	C12—C13—C14	119.61 (16)
C4—C3—C2	119.81 (14)	C12—C13—H13A	120.2
C4—C3—H3A	120.1	C14—C13—H13A	120.2
C2—C3—H3A	120.1	C15—C14—C13	119.77 (17)
C3—C4—C5	120.63 (14)	C15—C14—H14A	120.1
C3—C4—H4A	119.7	C13—C14—H14A	120.1
C5—C4—H4A	119.7	C14—C15—C16	121.40 (15)
C6—C5—C4	119.82 (14)	C14—C15—H15A	119.3
C6—C5—H5A	120.1	C16—C15—H15A	119.3
C4—C5—H5A	120.1	C15—C16—C17	119.44 (15)
C5—C6—C7	119.76 (13)	C15—C16—H16A	120.3
C5—C6—C8	121.34 (13)	C17—C16—H16A	120.3
C7—C6—C8	118.86 (13)	O2—C17—C12	115.80 (11)
C2—C7—C6	120.11 (13)	O2—C17—C16	125.05 (13)
C2—C7—H7A	119.9	C12—C17—C16	119.14 (13)
C6—C7—H7A	119.9	O2—C18—H18A	109.5
C6—C8—C9	112.34 (11)	O2—C18—H18B	109.5
C6—C8—H8A	109.1	H18A—C18—H18B	109.5
C9—C8—H8A	109.1	O2—C18—H18C	109.5
C6—C8—H8B	109.1	H18A—C18—H18C	109.5
C9—C8—H8B	109.1	H18B—C18—H18C	109.5

### Hydrogen-bond geometry (Å, °)

**Table:**

*D*—H···*A*	*D*—H	H···*A*	*D*···*A*	*D*—H···*A*
O3—H3···N3^i^	0.82	1.94	2.7569 (15)	173
C5—H5A···O1^ii^	0.93	2.59	3.406 (2)	147
C8—H8A···O2	0.97	2.57	3.485 (2)	157
C4—H4A···Cg3^iii^	0.93	3.25	4.004 (3)	140
C7—H7A···Cg3	0.93	3.16	4.067 (3)	165
C18—H18A···Cg2^iv^	0.96	3.03	3.400 (3)	105
C18—H18B···Cg2^iv^	0.96	3.08	3.400 (3)	101

Symmetry codes: (i) −*x*+2, −*y*, −*z*+2; (ii) −*x*+1/2, *y*−1/2, −*z*+3/2; (iii) *x*−1, *y*, *z*;  (iv) *x*−1/2, −*y*−1/2, *z*−3/2.
